# "A calorie is a calorie" violates the second law of thermodynamics

**DOI:** 10.1186/1475-2891-3-9

**Published:** 2004-07-28

**Authors:** Richard D Feinman, Eugene J Fine

**Affiliations:** 1 Department of Biochemistry, State University of New York Downstate Medical Center, Brooklyn, NY 11203 USA; 2 Department of Nuclear Medicine, Jacobi Medical Center, Bronx, NY 10461 USA

## Abstract

The principle of "a calorie is a calorie," that weight change in hypocaloric diets is independent of macronutrient composition, is widely held in the popular and technical literature, and is frequently justified by appeal to the laws of thermodynamics. We review here some aspects of thermodynamics that bear on weight loss and the effect of macronutrient composition. The focus is the so-called metabolic advantage in low-carbohydrate diets – greater weight loss compared to isocaloric diets of different composition. Two laws of thermodynamics are relevant to the systems considered in nutrition and, whereas the first law is a conservation (of energy) law, the second is a dissipation law: something (negative entropy) is lost and therefore balance is not to be expected in diet interventions. Here, we propose that a misunderstanding of the second law accounts for the controversy about the role of macronutrient effect on weight loss and we review some aspects of elementary thermodynamics. We use data in the literature to show that thermogenesis is sufficient to predict metabolic advantage. Whereas homeostasis ensures balance under many conditions, as a general principle, "a calorie is a calorie" violates the second law of thermodynamics.

## Review

The recent awareness of an epidemic of obesity coincides with, and may have contributed to a dramatic increase in the popularity of a variety of low carbohydrate diets. This rapid switch in dietary habits of a significant part of the population, and the virtual revolution in the food industry, is unusual in that it stands in direct opposition to long-standing recommendations of the majority of the nutritional and medical establishment (e.g. [1,2]). Despite isolated examples, such as a recent editorial by Walter Willet pointing to the need to understand low carbohydrate diets [3], there is still little real acceptance by nutrition professionals or health organizations. One aspect of these diets that has been especially controversial is the so-called metabolic advantage – the idea that more weight may be lost calorie for calorie compared with diets of higher carbohydrate content.

We recently reviewed the literature on metabolic advantage [4]. We showed that there is a sufficient number of reports in the literature to establish the existence of metabolic advantage and we tabulated results from ten or so studies demonstrating that low carbohydrate diets can lead to greater weight loss than isocaloric low fat diets. The reports we cited have frequently been met with the criticism that the data could not be right because they would violate the laws of thermodynamics ([5,6]). An example is the recent demonstration of metabolic advantage in a small, pilot study [7] which, despite its preliminary status, was extremely well controlled. Three groups were studied: A low carbohydrate group (LoCHO = 1800 kcal for men; 1500 kcal for women), a low fat group (LoFat, 1800 and 1500); a third group also consumed a low carbohydrate diet but an additional 300 kcalories were provided (LoCHO+300, 2100 and 1800). The order of average amount of weight lost was LoCHO = 23 lbs, LoCHO+300 = 20 lbs LoFat = 17 lbs. This work received a good deal of attention in the popular press. Media reports, however, included comments of experts that "It doesn't make sense, does it?" "It violates the laws of thermodynamics. No one has ever found any miraculous metabolic effects." ([5]). If this is an accurate quotation, it is odd indeed. Miraculous, or otherwise, a metabolic effect *was *found. In the absence of an identifiable methodological error, experimental data has to be accepted and numerous investigations, in fact, serve as precedents for Greene et al.'s findings (Reviews: [4,8]).

In our previous review of metabolic advantage [4] we showed that there is, in fact, no theoretical violation of the laws of thermodynamics, and we provided a plausible mechanism. In general the pathways for gluconeogenesis that are required in order to supply obligate glucose (e.g. to brain and CNS), in combination with increased protein turnover, could account for the missing energy. Here, we simplify the thermodynamic argument and review some of the relevant principles. We show, moreover, that well-established data in the traditional nutritional literature predict metabolic advantage and no one should be surprised. The ironic conclusion is that the principle that weight gain on isocaloric diets must *always *be independent of macronutrient composition would violate the second law of thermodynamics.

## What do we mean by "a calorie is a calorie?"

Because it is a colloquial phrase, it is important to understand exactly what it is meant by "a calorie is a calorie." The most common meaning is that is it impossible for two isocaloric diets to lead to different weight loss. Frequently, the concept is justified by reference to the "laws of thermodynamics", but an explicit connection has never been spelled out. More recently, Buchholz & Schoeller [10] appear to identify "a calorie is a calorie" with the first law of thermodynamics. They also admit that high protein /low carbohydrate diets can lead to greater weight loss than isocaloric low fat diets in agreement with our assessment [4]. Nonetheless they maintain that "a calorie is a calorie," now justifying it by their connection of the phrase to the concept of energy conservation. It is important to point out that no study of isocaloric diets has ever claimed that the first law of thermodynamics is not true. Buchholz & Schoeller [10] have limited themselves by only including the first law and, therefore, do not understand how the differential weight loss could occur and think it "deserves further study." Our major point here is that there is more than one law of thermodynamics and that a more accurate understanding of the role of the second law shows that differential weight loss is not inconsistent with any physical principle.

## Thermodynamics

The idea that "a calorie is a calorie" comes from a misunderstanding of the laws of thermodynamics. There are two laws of thermodynamics. (The zeroth law that establishes the concept of temperature and the third law that describes absolute zero are not relevant here). When speaking of "the law*s *of thermodynamics" it is important to be sure that one is including the second law. The first law is very different in character from the second law [9,11,12]. The first law is a conservation law: it says that the form of energy may change, but the total is always conserved. The second law is a *dissipation *law: it defines a quantity, the entropy, S, which we traditionally identify with disorder or high probability. The second law says that in any (real) irreversible process, the entropy must increase (ΔS > 0); balance is *not *expected. Entropy is, in fact, identifiable with irreversibility.

It is important to understand that it is the second law that drives chemical reactions. The first law is a bookkeeping law and tells us that the total energy attributed to work, heat and changes in chemical composition will be constant. It does not tell us whether such a reaction will occur, or if it does, what the relative distributions of the forms of energy will be. To predict the tendency of the reaction to occur, we must employ the second law that says the entropy must increase. In a chemical reaction, at constant temperature and pressure, the entropic and energetic effects are combined into the change in the Gibbs free energy, ΔG, whose sign predicts the direction of reaction, and whose magnitude indicates the maximum amount of work realizable from the reaction.

## Application of ΔG'

**Figure 1:**
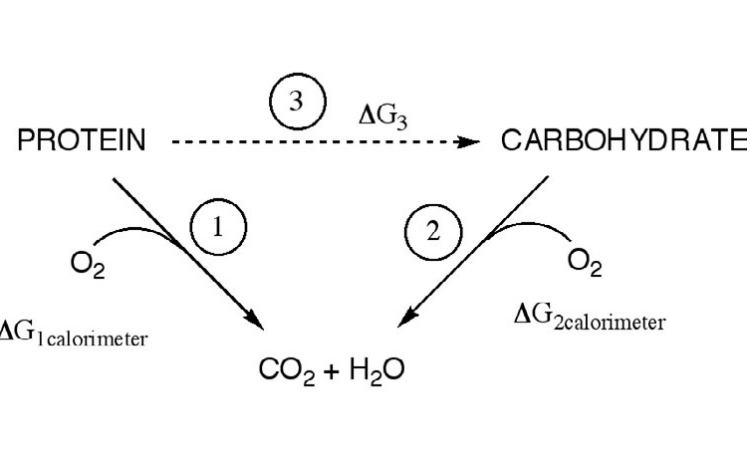
Pathways for oxidation of macronutrients.

To understand the implications of "a calorie is a calorie," that energy yield could be path-independent and the same for all diets consider that it implies that carbohydrate and protein are equivalent fuels as shown in Figure 1. The diagram indicates that, because it is a state variable, the free energy (ΔG') for Path 1 must be equal to that for path 2 + 3. If the ΔG' values for path 1 and path 2 are taken to be their calorimeter values, they will be approximately equal (~4 kcal/g, path 1 corrected for ureagenesis). This means that ΔG' for path 3, the conversion of protein to carbohydrate (also corrected) must be about zero. There exists at least one condition where this is not true, the standard state; it is generally considered that gluconeogenesis from one mole of alanine requires about 6 ATP [13,14]. Of course free energies are concentration dependent, so *in vivo *values will differ from standard state values but they are continuous functions of the concentrations and there will be numerous conditions under which ΔG' is not zero. In other words, assuming that protein and carbohydrate are energetically equivalent leads to a contradiction.

## Inefficiency

The second law was developed in the context of the industrial revolution and the attempt to understand the efficiency of machines. The law describes the theoretical limits on the efficiency of engines and applies as well to living (irreversible) systems. The second law says that no machine is completely efficient. Some of the available energy is lost as heat and in the internal rearrangement of chemical compounds and other changes in entropy. In other words, although the first law holds even in irreversible processes – energy is still conserved – the second law says that something is lost, something *is *unrecoverable. The efficiency of a machine is dependent on how the machine works and, for a biochemical machine, the nature of the fuel and the processes enlisted by the organism. A simple example is the inefficiency of low-test gasoline in high compression gasoline engines. If a "calorie is a calorie" were true, nobody would pay extra for high test gasoline. (The calorimeter values of a gasoline will be the same whether or not it contains an antiknock compound). In weight loss diets, of course, inefficiency is desirable and is tied to hormonal levels and enzyme activities

## Efficiency and thermogenesis

**Figure 2:**
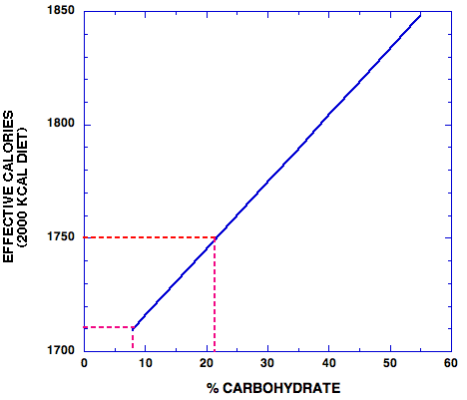
The dependence of effective calories on % carbohydrate in a 2000 kcal diet. Effective calories were determined by subtracting the losses due to thermogenesis as described in the text.

In nutrition, one component of inefficiency is measured in thermogenesis (thermic effect of feeding), or the heat generated in processing food. There is a large literature on this subject and the general conclusion, as summarized in a recent review by Jéquier [15], is that thermic effects of nutrients is approximately 2–3 % for lipids, 6–8 % for carbohydrates, and 25–30% for proteins. It is interesting that this data itself might be enough to explain metabolic advantage. Here we took the average of Jéquier's values (2.5, 7 and 27.5 % for fat, CHO and protein) and calculated the effective energy yield for a 2000 kcal diet. If we assume a diet composition of CHO:fat: protein of 55:30:15, within the range of commonly recommended diets, the calculated effective yield is 1848 kcal. We now consider the effect of reducing carbohydrate progressively and substituting the calories removed equally between fat and protein. Figure 2 shows that the wasted calories due to thermogenesis increase as carbohydrate is reduced and reach 100 kcal at 21 % carbohydrate. This value of 100 kcal is recommended by several professionals as the goal for daily weight reduction (e.g. [16]). Notably, at 8 % CHO, the value for the early phase of the Atkins [17], South Beach [18] or Protein Power diets [19], 140 kcalories are lost as heat. Now, there will be metabolic accommodations and one can't predict that the ratios will stay the same over a long term diet, but the calculations show that the *possibility *of metabolic advantage should not come as a surprise.

Recommendations for fighting obesity frequently call for small reductions in calories [16]. In fact, given the resistance of steady state systems to small perturbations it is doubtful that this is a promising strategy. Nonetheless, taking the goal at face value, if it could be achieved by a simple change in macronutrient composition, such a method would seem worthy of serious consideration. The arguments above show that such a phenomenon is possible. There are plausible arguments for how it could take place and substantial experimental evidence for its occurrence [4].

## Conclusions

A review of simple thermodynamic principles shows that weight change on isocaloric diets is not expected to be independent of path (metabolism of macronutrients) and indeed such a general principle would be a violation of the second law. Homeostatic mechanisms are able to insure that, a good deal of the time, weight does not fluctuate much with changes in diet – this might be said to be the true "miraculous metabolic effect" – but it is subject to many exceptions. The idea that this is *theoretically *required in all cases is mistakenly based on equilibrium, reversible conditions that do not hold for living organisms and an insufficient appreciation of the second law. The second law of thermodynamics says that variation of efficiency for different metabolic pathways *is to be expected*. Thus, ironically the dictum that a "calorie is a calorie" *violates the second law of thermodynamics*, as a matter of principle.

The analysis above might be said to be over-kill although it is important, intellectually, not to invoke the laws of thermodynamics inappropriately. There are also, however, practical consequences. The seriousness of the obesity epidemic suggests that we attack it with all the means at our disposal. Metabolic advantage with low carbohydrate diets is well established in the literature. It does not always occur but the important point is that it can occur. To ignore its possibilities and to not investigate the precise conditions under which it appears would be cutting ourselves off from potential benefit. The extent to which metabolic advantage will have significant impact in treating obesity is unknown and it is widely said in studies of low carbohydrate diets that "more work needs to be done." However, if the misconception is perpetuated that there is a violation of physical laws, that work will not be done, and if done, will go unpublished due to editorial resistance. Attacking the obesity epidemic will involve giving up many old ideas that have not been productive. "A calorie is a calorie" might be a good place to start.

## Competing interests

None declared.
